# Supplementary material for: Adult stem cells and niche cells segregate gradually from common precursors that build the adult Drosophila ovary during pupal development
Source: eLife. 2021 Sep 30;10:e69749. doi: 10.7554/eLife.69749 (PMC8536258; doi:10.7554/eLife.69749)
Supplement: Transparent reporting form [file elife-69749-transrepform1.docx]

***eLife’s* transparent reporting form**

We encourage authors to provide detailed information *within their submission* to facilitate the interpretation and replication of experiments. Authors can upload supporting documentation to indicate the use of appropriate reporting guidelines for health-related research (see [EQUATOR Network](http://www.equator-network.org/%20)), life science research (see the [BioSharing Information Resource](https://biosharing.org/)), or the [ARRIVE guidelines](http://www.plosbiology.org/article/info:doi/10.1371/journal.pbio.1000412) for reporting work involving animal research. Where applicable, authors should refer to any relevant reporting standards documents in this form.

If you have any questions, please consult our Journal Policies and/or contact us: [editorial@elifesciences.org](mailto:editorial@elifesciences.org).

**Sample-size estimation**

- You should state whether an appropriate sample size was computed when the study was being designed
- You should state the statistical method of sample size computation and any required assumptions
- If no explicit power analysis was used, you should describe how you decided what sample (replicate) size (number) to use

Please outline where this information can be found within the submission (e.g., sections or figure legends), or explain why this information doesn’t apply to your submission:

For lineage analyses scored in adults, we aimed to score at least 50 informative ovarioles for each condition. This objective was based on prior experience analyzing lineages initiated in adult ovaries and represents a compromise between a sufficient size to account for variability and practical issues limiting sample size (time spent collecting and scoring images). Sample size (n) is displayed in each graph or table where relevant.

**Replicates**

- You should report how often each experiment was performed
- You should include a definition of biological versus technical replication
- The data obtained should be provided and sufficient information should be provided to indicate the number of independent biological and/or technical replicates
- If you encountered any outliers, you should describe how these were handled
- Criteria for exclusion/inclusion of data should be clearly stated
- High-throughput sequence data should be uploaded before submission, with a private link for reviewers provided (these are available from both GEO and ArrayExpress)

Please outline where this information can be found within the submission (e.g., sections or figure legends), or explain why this information doesn’t apply to your submission:

Information about biological replicates (number of ovarioles evaluated) is available in methods, graphs and figure legends. Ovarioles from individual animals were mounted on separate slides, allowing us to look at variation between animals but in the reported experiments there were no marked systematic differences, so the results from all ovarioles, derived from several animals, were presented collectively. Scoring of samples provides a definitive numerical result of the number of marked cells in specific locations. Samples where this was not possible because of poor staining or images were not included. Many samples were re-scored by a second experimenter, followed by discussions, to ensure a uniform standard. Each set of experiments of a certain type (presented in a single Figure, for example) was scored entirely by one individual to ensure uniform standards (and some parts were scored by a second experimenter to ensure uniform standards among all sets of experiments).

**Statistical reporting**

- Statistical analysis methods should be described and justified
- Raw data should be presented in figures whenever informative to do so (typically when N per group is less than 10)
- For each experiment, you should identify the statistical tests used, exact values of N, definitions of center, methods of multiple test correction, and dispersion and precision measures (e.g., mean, median, SD, SEM, confidence intervals; and, for the major substantive results, a measure of effect size (e.g., Pearson's r, Cohen's d)
- Report exact p-values wherever possible alongside the summary statistics and 95% confidence intervals. These should be reported for all key questions and not only when the p-value is less than 0.05.

Please outline where this information can be found within the submission (e.g., sections or figure legends), or explain why this information doesn’t apply to your submission:

N values, means and SEMs are presented in relevant graphical data. The results do not address different behaviors due to altered genotypes, for example, so that statistical tests of different outcomes are not generally relevant. Several sets of results report the different types of marked cell found in single ovarioles. The mathematical approaches to estimate the frequency of instances where marked cells derive from a single original marked cell, and the consequent estimation of single-cell lineage frequencies of different types are described in Materials & Methods.

(For large datasets, or papers with a very large number of statistical tests, you may upload a single table file with tests, Ns, etc., with reference to sections in the manuscript.)

**Group allocation**

- Indicate how samples were allocated into experimental groups (in the case of clinical studies, please specify allocation to treatment method); if randomization was used, please also state if restricted randomization was applied
- Indicate if masking was used during group allocation, data collection and/or data analysis

Please outline where this information can be found within the submission (e.g., sections or figure legends), or explain why this information doesn’t apply to your submission:

The nature of each group is defined solely by timing characteristics and is explained in the main text, all relevant figures and Materials and Methods.

**Additional data files (“source data”)**

- We encourage you to upload relevant additional data files, such as numerical data that are represented as a graph in a figure, or as a summary table
- Where provided, these should be in the most useful format, and they can be uploaded as “Source data” files linked to a main figure or table
- Include model definition files including the full list of parameters used
- Include code used for data analysis (e.g., R, MatLab)
- Avoid stating that data files are “available upon request”

Please indicate the figures or tables for which source data files have been provided:

A source data document containing numerical values used in all graphs will be provided.
